# Quality of life in children with Down syndrome and its association with parent and child demographic characteristics: Parent‐reported measures

**DOI:** 10.1002/mgg3.2337

**Published:** 2023-12-13

**Authors:** Nuha Alrayes, Noha M. Issa, Omar Y. Alghubayshi, Jumana Y. Al‐Amaa, Ashwaq Hassan Alsabban, Dalal Sameer Al Shaer, Reem Abdullah Alyoubi, Khalidah K. Nasser, Yaser M. Alkhiary

**Affiliations:** ^1^ Department of Medical Laboratory Sciences, Faculty of Applied Medical Sciences King Abdulaziz University Jeddah Saudi Arabia; ^2^ Princess Al‐Jawhara Center of Excellence in Research of Hereditary Disorders King Abdulaziz University Jeddah Saudi Arabia; ^3^ Department of Genetic Medicine, Faculty of Medicine King Abdulaziz University Jeddah Saudi Arabia; ^4^ Department of Human Genetics, Medical Research Institute Alexandria University Alexandria Egypt; ^5^ Saudi Arabia Ministry of Health, Khobar Hospital and Health Centers Management Al‐Khobar Saudi Arabia; ^6^ Department of Biological Science, Faculty of Sciences King Abdulaziz University Jeddah Saudi Arabia; ^7^ Pediatric Department King Abdulaziz University Hospital Jeddah Saudi Arabia; ^8^ Oral and Maxillofacial Prosthodontics Department, Faculty of Dentistry King Abdulaziz University Jeddah Saudi Arabia

**Keywords:** demographic data, Down syndrome, KIDSCREEN‐27, quality of life

## Abstract

**Background:**

This study aims to explore the association between the quality of life (QoL) in children with Down syndrome (DS) and its relationship with demographic characteristics of both parents and children. The investigation encompasses five domains: physical and psychological well‐being, autonomy and parental relationship, social well‐being, and peers, as well as school and the learning environment.

**Method:**

An online questionnaire, the KIDSCREEN‐27, was used to measure the QoL of 112 families with DS in Saudi Arabia, referred to as “Parent‐Reported Measures.” Descriptive statistics were analyzed using the Statistical Package for Social Sciences.

**Results:**

The study found that the QoL of children with DS showed high scores in the psychological well‐being, autonomy, parental relations, school, and learning environment domains. However, the physical and social well‐being and peer domains had lower scores, although still considered “good scores.” Family income had a positively significant influence on all QoL domains. Specifically, higher family income was associated with better QoL outcomes, except for social well‐being. Parental age was found to influence psychological well‐being, while parental education and the relationship between the parent and child influenced social well‐being. Lastly, the child's gender was found to have an impact on the school and learning environment domain.

**Conclusion:**

The study highlights the importance of understanding the impact of the demographic variability of children with DS and their parents on the QoL of their children. It emphasizes the need to address the needs of families with lower incomes and the importance of parental education and relationships with their children in improving social well‐being. The findings could aid policymakers and healthcare providers in improving the QoL for families with children who have DS.

## Abstract 2

This study aims to determine the relationship between demographic variables of parents with down syndrome (DS) children and their quality of life (QoL). It is the first time to use the Arabic version KIDSCREEN questionnaire, after validation. This is the first study to measure the demographic variables influence on the reported QoL of children with DS. This study found that family income influenced all QoL domains except social well‐being. Psychological well‐being was influenced by parental age. Social well‐being was influenced by parental education. Finally, the school and learning environment was influenced by the child's gender.

## 1 INTRODUCTION

Down syndrome (DS) is a genetic condition caused by the presence of an extra copy of chromosome 21, also known as “Trisomy 21” (Frederiksen et al., 2018). The condition is associated with intellectual disability and developmental delays, making it a leading cause of these conditions in children (Grieco et al., 2015). Maternal age is a well‐known risk factor for having a child with DS, with the risk increasing as women get older, especially in women over 40 (Frederiksen et al., 2018).

DS is linked to various chromosomal causes with morphological, biochemical, and physiological consequences (Alaama et al., 2015). In addition to intellectual disability and developmental delays, DS is associated with congenital cardiac abnormalities in more than 50% of newborns, an increased risk of both congenital and acquired thyroid disease, and a higher incidence of conditions such as hearing loss, diabetes, leukemia (Al‐Aama et al., 2012; AlAaraj et al., 2019; Bergström et al., 2016; Manickam et al., 2016), obesity, gastrointestinal disorders, and obstructive sleep apnea syndrome (Basil et al., 2016; Lagan et al., 2020; Saadah et al., 2012). Individuals with DS show varying levels of intellectual disability, and Alzheimer's disease is a significant concern among adults with the condition (Fortea et al., 2021). As per American Academy of Pediatrics (AAP) guidelines (Bull & Committee on Genetics, 2011), children with DS require comprehensive care, including prenatal, natal, and postnatal support. Prospective mothers aged over 35 should also receive genetic counseling (GC) sessions to understand the associated risks and available support services (Aldhwayan et al., 2015; Schwartz & Vellody, 2016).

From the 1960s onwards, the significance of quality of life (QoL) has been a subject of debate in medical literature. With increasing life expectancy and medical advancements, its importance has grown. While various definitions of QoL highlight subjective elements such as life satisfaction, others emphasize more objective aspects like general well‐being (Karimi & Brazier, 2016). The 1948 definition of health by the World Health Organization (WHO) encompasses the concept of QoL as not solely the absence of disease but also the presence of physical, mental, and social well‐being, along with a harmonious relationship with the environment (Karimi & Brazier, 2016).

Personality traits and qualities that can be influenced by a person's activities or surroundings are referred to as demographic factors. The impacts of family income level and socioeconomic status on QoL have received a lot of attention lately (Currie, 2020). For instance, family income, ethnicity, and education level can affect the level of intervention and the child's overall QoL (Assari & Caldwell, 2019; Mohammed Nawi et al., 2013).

This study aims to provide valuable insights into the factors that could affect the QoL of children with DS in SA and could guide the development of interventions and policies to enhance their well‐being. Genetic counselors in SA also face challenges in providing appropriate counseling and support to families of children with DS due to the scarcity of literature on the QoL of children with DS in the country. However, it is essential to acknowledge that the results may not be generalizable to other countries due to the diversity of cultural, social, and economic factors that may influence the lives of individuals with DS. Therefore, further research is necessary to explore effective strategies to promote the QoL of people with DS in various global contexts. This study's findings are crucial for genetic counselors and other healthcare professionals who provide care and support to families of children with DS, ultimately improving the QoL of children with DS in SA.

In his study, differences in demographics between children with DS and their parents were hypothesized to be linked to differences in the DS children's QoL. The study used the KIDSCREEN‐27 questionnaire, which is a well‐known, reliable tool, used in more than 37 countries around the world. The original Arabic version of the questionnaire was requested from its source and used after it was validated on the Saudi population. The study assessed the instrument's validity through Pearson's correlation coefficient and confirmed it using the validity factor of Cronbach's alpha (*α*) and Bartlett's test. To the best of our knowledge, this is the first attempt to validate the Arabic version of the KIDSCREEN‐27 on the Saudi population. Additionally, as far as known, this is the first study to measure the demographic variables on the reported QoL of children with DS. Upon reviewing the literature, studies that focused on this topic were lacking. Accordingly, this study aims to detect statistically significant effects in the link between differences in the QoL of children with DS and the demographic variables of these children and their parents.(e.g., parental age, parental education level, nationality, income, relationship to the child, and child's gender) by quantifying the range of reported QoL for children with DS in five domains: physical well‐being; psychological well‐being; autonomy and parent relationships; social well‐being and peers; school and learning environment.

## 2 METHODOLOGY

### 2.1 Ethical compliance

The ethical approval for this study was filed with the institutional review board at the King Abdulaziz University Hospital (KAUH) in Jeddah after the preparation of the research proposal. The study was officially approved by the biomedical ethics and research committee at the King Abdulaziz University, Jeddah, Saudi Arabia (SA) (Reference No. 257‐20). Informed consent forms accompanied each version of the questionnaire. Each participant was informed about the study, provided with the researcher's contact information, provided with information about confidentiality, how their data would be used and assured that they had the choice to participate or not before attempting to answer the first question.

### 2.2 Recruitment

The present study aims to determine the relationship between quality of life (QoL) in children with DS and its association with parent and child demographic characteristics across five domains: physical and psychological well‐being, autonomy and parental relationship, social well‐being and peers, and school and the learning environment. The study was prompted by clinical observations made by genetic counselors working at a DS clinic at KAUH, in Jeddah, SA. This is a tertiary care teaching hospital in Jeddah, the second largest city in the Kingdom at the Mecca administrative region that holds a population of 6,915,006 (General Authority for Statistics, Kingdom of Saudi Arabia, 2010). The researchers hypothesize that the reported QoL of children with DS in SA is satisfactory, but that demographic variables, such as parental age, education level, nationality, income, relation to the child, and gender of the DS child, significantly affect their QoL. To assess these hypotheses, the researchers have formulated two research questions. The first research question seeks to determine the range of reported QoL of children with DS in five domains, including physical and psychological well‐being, autonomy and parental relations, social well‐being and their peers, the school, and the learning environment. The second research question aims to investigate whether there are statistically significant differences in the QoL of children with DS that can be attributed demographic variables of these children and their parents. The study is cross‐sectional in nature and employs multiple approaches to analyze the data. The researchers aim to detect statistically significant effects of demographic variables on the QoL of children with DS, highlighting individual differences to guide genetic counselors and other healthcare professionals in tailoring practices to effectively meet the needs of parents and children while enhancing their QoL.

### 2.3 Data collection method

A questionnaire was disseminated online through the Ministry of Education database, the Genetics Clinic at KAUH, and several social media groups on WhatsApp and Twitter, for parents of one or more children with DS in school (6–18 years) in SA. If a Caregiver who was not a parent or not living in the country, that person was excluded.

### 2.4 Study tool

The Arabic version of KIDSCREEN‐27 questionnaire (Appendix S1) was used for this study. The questionnaire contains 27 statements that respond to the five domains, including physical and psychological well‐being, autonomy and parental relations, social well‐being and their peers, the school, and the learning environment. Each participant specifies their response for each statement by selecting one of the five classifications excellent, very good, good, fair, and poor. Questions about demographic data of both the child and parent were included in the questionnaire (KIDSCREEN Group, n.d.).The demographic data collected for parents included their relation to the child (mother, father or parent), age (less than 30 years, from 30 to 40 years, more than 40 years), nationality (Saudi or non‐Saudi), location of residence (Jeddah City, Mecca Province (excluding Jeddah City), Al‐Madinah Province, Al Riyadh Province, Eastern Province, Al‐Qassim Province, Asser Province, Northern Borders Province), education level (less than primary, primary, intermediate (Prep.), secondary, bachelor, postgraduate). And Total financial status of family (in Saudi Arabian Riyals), SAR 3000 or less, SAR 3001–6000, SAR 6001–10,000, more than SAR 10,000.

The demographic data collected for children with DS included the number of children with DS in the family (1 child, 2 children, 3 children), gender (female or male), age (6–18 years old), and the maternal age when the child with DS was born were also collected.

### 2.5 Validity and reliability of the original Arabic KIDSCREEN‐27 questionnaire version in the Saudi population

#### 2.5.1 Validating internal consistency

To verify internal consistency of the questionnaire, the Pearson's correlation coefficient was calculated to determine the degree of correlation of each of the questionnaire expressions to the overall degree of the axis to which the item belongs on for 112 participants' data.

#### 2.5.2 Validity of the study tool

The study instrument validity was confirmed by using the validity factor of Cronbach's alpha (*α*), that is widely used measure of internal consistency or reliability of a survey instrument and Bartlett's test (112) for 112 participants' data.

### 2.6 Data analysis

Table 1 shows mean values and corresponding QoL to be calculated for each domain. More specifically, percentages, arithmetic mean, and standard deviations for five domains will assess the range of QoL (poor, fair, good, very good, and excellent) based on the arithmetic mean of expressions and the weighted mean of the domain (Table 1), which answer the first question.

**TABLE 1:** Mean value and corresponding ranges of QoL.

Section	Range of quality of life	Minimum	Maximum	Considered value
1	Poor	1	1 + 0.8 = 1.8	1.8 or less
2	Fair	>1.8	>1.8 + 0.8 = 2.6	>1.8 to 2.6
3	Good	>2.6	2.6 + 0.8 = 3.4	>2.6 to 3.4
4	Very good	>3.4	3.4 + 0.8 = 4.2	>3.4 to 4.2
5	Excellent	>4.2	4.2 + 0.8 = 5	>4.2 to 5

Based on the weighted mean approach, the Likert scale's fifth range in the KID SCREEN‐27 questionnaire has been divided into five distinct sections, as outlined below:

1. The scale's minimum value was 1, denoting “not at all” or “never,” while the maximum value was 5, indicating “extremely” or “always.” Consequently, the range equates to 4 (5–1).
2. To evenly segment the responses into five parts, the range was divided by 5, resulting in 0.8.
3. The value of 0.8 was established as the equal interval between the five sections, leading to the following divisions:

This table shows the range of QoL and their corresponding mean values. A QoL score of less than 1.8 is considered poor, while a score from 1.8 to 2.6 is considered fair. A score from 2.6 to 3.4 is considered good, while a score from 3.4 to 4.2 is considered very good. A score above 4.2 is considered excellent.

The second research question requires a detailed examination of the statistical effect of each demographic variable on five QoL domains. Statistical Package for Social Sciences software was used for statistical analysis of descriptive statistics, including the mean, standard deviation, and correlation. Mann–Whitney and Kruskal–Wallis tests evaluated differences in QoL among DS children that could be attributed to these children and their parents' demographic variables. In all tests, a *p*‐value of less than 0.05 indicated statistical significance. The Mann–Whitney test examined “differences between two groups on a single, ordinal variable with no specific distribution” (MacFarland & Yates, 2016), while the Kruskal–Wallis (one way analysis of variance) “test among three or more independently sampled groups on a single, non‐normally distributed continuous variable” could examine more (McKnight & Najab, 2010).

## 3 RESULTS

### 3.1 Validity and reliability of the original Arabic KIDSCREEN‐27 questionnaire in the Saudi population

#### 3.1.1 Validating internal consistency

To verify the internal consistency of the questionnaire, Pearson's correlation coefficient was calculated to determine the degree of correlation of each of the questionnaire expressions to the overall degree of the axis/domain to which the phrases/questions belongs. The following tables show the correlation coefficients for each of the axes including the terms of expression. The total degree of the dimension is significant at the level of significance *α* ≤ 0.05.

Table 2 indicates that the values of the correlation coefficient for each of the phrases/questions with their dimension are positive and statistically significant at the level of significance *α* ≤ 0.05. This indicates that the internal consistency in this axis is valid.

**TABLE 2:** Pearson correlation coefficient for first‐axis (physical well‐being) expressions with the overall grade of the axis/112 participants.

The first axis(physical well‐being domain)			
Number	Phrases/questions	Correlation coefficient	Sig.
1	In general, how would your child rate her/his health?	0.768**	<0.01
2	Has your child felt fit and well?	0.717**	<0.01
3	Has your child been physically active?	0.801**	<0.01
4	Has your child been able to run well?	0.862**	<0.01
5	Has your child felt full of energy?	0.766**	<0.01

** Correlation is significant at the ≤0.05 level.

Table 3 indicates that the values of the correlation coefficient for each of the items with their dimension are positive and statistically significant at the level of significance *α* ≤ 0.05. This indicates that the internal consistency in this axis is valid.

**TABLE 3:** Pearson correlation coefficient for second‐axis (psychological well‐being) expressions with the overall grade of the axis/112 participants.

The second axis (psychological well‐being domain)			
Item number	Item	Correlation coefficient	Sig.
1	Has your child felt that life was enjoyable?	0.744**	<0.01
2	Has your child been in a good mood?	0.738**	<0.01
3	Has your child had fun?	0.803**	<0.01
4	Has your child felt sad?	0.665**	<0.01
5	Has your child felt so bad that he/she did not want to do anything?	0.630**	<0.01
6	Has your child felt lonely?	0.785**	<0.01
7	Has your child been happy with the way he/she is?	0.771**	<0.01

** Correlation is significant at the ≤0.05 level.

Table 4 indicates that the values of the correlation coefficient for each of the items with their dimension are positive and statistically significant at the level of significance *α* ≤ 0.05. This indicates that the internal consistency in this axis is valid.

**TABLE 4:** Pearson correlation coefficient for third‐axis (autonomy and parent relation domain) expressions with the overall grade of the axis/112 participants.

The third axis (autonomy and parent relation)			
Number	Phrases/questions	Correlation coefficient	Sig.
1	Has your child had enough time for him/herself?	0.509**	<0.01
2	Has your child been able to do the things that he/she wants to do in his/her free time?	0.630**	<0.01
3	Has your child felt that his/her parent(s) had enough time for him/her?	0.506**	<0.01
4	Has your child felt that his/her parent(s) treated him/her fairly?	0.622**	<0.01
5	Has your child been able to talk to his/her parent(s) when he/she wanted to?	0.464**	<0.01
6	Has your child had enough money to do the same things as his/her friends?	0.761**	<0.01
7	Has your child felt that he/she had enough money for his/her expenses?	0.741**	<0.01

** Correlation is significant at the ≤0.05 level.

Table 5 indicates that the values of the correlation coefficient for each of the items with their dimension are positive and statistically significant at the level of significance *α* ≤ 0.05. This indicates that the internal consistency in this axis is valid.

**TABLE 5:** Pearson correlation coefficient for fourth‐axis (social well‐being and peers) expressions with the overall grade of the axis/112 participants.

The fourth axis (social well‐being and peers domain)			
Number	Phrases/questions	Correlation coefficient	Sig.
1	Has your child spent time with his/her friends?	0.866**	<0.01
2	Has your child had fun with his/her friends?	0.900**	<0.01
3	Have your child and his/her friends helped each other?	0.898**	<0.01
4	Has your child been able to rely on his/her friends?	0.828**	<0.01

** Correlation is significant at the ≤0.05 level.

Table 6 indicates that the values of the correlation coefficient for each of the items with their dimension are positive and statistically significant at the level of significance *α* ≤ 0.05. This indicates that the internal consistency in this axis is valid.

**TABLE 6:** Pearson correlation coefficient for fifth‐axis (school and learning) expressions with the overall grade of the axis/99 participants.

The fifth axis (school and learning)			
Number	Phrases/questions	Correlation coefficient	Sig.
1	Has your child been happy at school?	0.807**	<0.01
2	Has your child got on well at school?	0.892**	<0.01
3	Has your child been able to pay attention?	0.686**	<0.01
4	Has your child got along well with his/her teachers?	0.617**	<0.01

** Correlation is significant at the ≤0.05 level.

#### 3.1.2 Validity of the study tool

The study instrument validity was confirmed by using the validity factor of Cronbach's alpha (α) and Bartlett's test.

The following table indicates that the general validity coefficient is high. It measured 0.869 for Cronbach's alpha and 0.707 for Bartlett's test. This indicates that the questionnaire has a high degree of validity and can be relied on in this study (Table 7).

**TABLE 7:** Cronbach alpha and Bartlett's test coefficient to measure the validity of the study instrument.

Axes/domain of the questionnaire	*N*	Cronbach's alpha	Bartlett's test
Physical well‐being	5	0.843	0.804
Psychological well‐being	7	0.857	0.829
Autonomy and parent relation	7	0.714	0.586
Social well‐being and peers	4	0.896	0.751
School and learning	4	0.749	0.586
General constancy	27	0.869	0.707

In Table 8, the number of participating mothers (76.8%) was three times more than the number of participating fathers (23.2%). The majority were Saudi (86.6%), versus non‐Saudi (13.4%), living in Jeddah (35.7%), followed by those living in the Eastern Province (17.9%), Riyadh (17%), Al‐Madinah (16.1%), Mecca (8.9%), Al‐Qassim and Asser (1.8%), and Northern Borders (0.9%). The majority attended college/Bachelor (59.8%), followed by those with Secondary education (22.3%), postgraduate and intermediate (Prep.) (8%), and Primary and less than Primary (0.9%). The financial status of participants was distributed across the spectrum SAR 3000 or less (7.1%), SAR 3001–6000 (22.3%), SAR 6001–10,000 (25%), and more than 10,000 (45.5%).

**TABLE 8:** Demographic data of participants.

	Count	%
Relation to child		
Mother	86	76.8
Father	26	23.2
Age		
Less than 30 years	21	18.8
From 30 to 40 years	23	20.5
More than 40 years	68	60.7
Nationality		
Saudi	97	86.6
Non‐Saudi	15	13.4
Location of residence		
Jeddah city	40	35.7
Mecca Province (excluding Jeddah City)	10	8.9
Al‐Madinah Province	18	16.1
Al Riyadh Province	19	17.0
Eastern Province	20	17.9
Al‐Qassim Province	2	1.8
Asser Province	2	1.8
Northern Borders Province	1	0.9
Education level		
Less than primary	1	0.9
Primary	1	0.9
Intermediate (prep.)	9	8.0
Secondary	25	22.3
Bachelor	67	59.8
Postgraduate	9	8.0
Financial status (Saudi Arabian Riyal)		
SAR 3000 or less	8	7.1
SAR 3001–6000	25	22.3
SAR 6001–10,000	28	25.0
More than SAR 10,000	51	45.5

As shown in Table 9, of the 112 DS children, 47.3% were female, while 52.7% were male. 96.4% of the children were the only child with DS in the family, while 1.8% had another DS member of the family and another 1.8% had two members of the family with DS. The most frequent child with DS aged 8 years (15.2%), 6 years (14.3%), 7 years (10.7%), 9 years (8%), 10, 13, and 15 years (7.1%), 16 years (6.3%), 18 years (5.4%), 11 and 17 years (4.5%), and 12 years (3.6%). 30.4% of the mothers of the DS children were between the ages of 31 and 40 years at conception, 23.2% were 30 years or less, and 16.1% were 41 years old or more.

**TABLE 9:** Personal characteristics of the child with DS.

	Count	%
Number of children with DS in the family		
1 child	108	96.4
2 children	2	1.8
3 children	2	1.8
Child's gender		
Female	53	47.3
Male	59	52.7
Child's age		
6 years	16	14.3
7 years	12	10.7
8 years	17	15.2
9 years	9	8.0
10 years	8	7.1
11 years	5	4.5
12 years	4	3.6
13 years	8	7.1
14 years	7	6.3
15 years	8	7.1
16 years	7	6.3
17 years	5	4.5
18 years	6	5.4
Maternal age when the child with DS was born		
30 years or less	26	23.2
31–35 years	34	30.4
36–40 years	34	30.4
41 years or more	18	16.1

##### First domain: Physical well‐being

Table 10 shows that the reported QoL of the children with DS on the physical well‐being domain is “good” with an average of (3.36 out of 5.00). This average falls in the third category of fifth scale categories in the scale of weighted mean for QoL table (from 2.6 to 3.4).

**TABLE 10:** Range of reported QoL in the domain of physical well‐being.

Question	Count	%	Mean	Std. deviation	Rank	Range
In general, how would your child rate her/his health?						
Weak	2	1.8	3.7679	1.03960	1	Very good
Acceptable	11	9.8				
Good	31	27.7				
Very good	35	31.3				
Excellent	33	29.5				
Has your child felt fit and well?						
Not at all	0	0.0	3.5268	0.94878	2	Very good
Yes, a little	17	15.2				
Yes, average	38	33.9				
Yes, very much	38	33.9				
Yes, enough	19	17.0				
Has your child been physically active?						
Not at all	8	7.1	3.0179	1.03960	4	Good
Yes, a little	27	24.1				
Yes, average	40	35.7				
Yes, very much	29	25.9				
Yes, enough	8	7.1				
Has your child been able to run well?						
Not at all	8	7.1	2.9911	1.04404	5	Good
Yes, a little	30	26.8				
Yes, average	36	32.1				
Yes, very much	31	27.7				
Yes, enough	7	6.3				
Has your child felt full of energy?						
Not at all	2	1.8	3.4911	0.93958	3	Very good
Yes, rarely	12	10.7				
Yes, sometimes	44	39.3				
Often yes	37	33.0				
Yes, always	17	15.2				
Physical well‐being			3.3589	0.78643	Good	

The responses also indicated that the first question, “In general, how would your child rate her/his health?”, scored the highest in terms of QoL with an average of (3.76 out of 5). On the other hand, the fourth question, “Has your child been able to run well?”, scored the least in terms of QoL with an average of (2.99 out of 5).

##### Second domain: Psychological well‐being

Data in this domain is approached with same previous procedure of data in the previous domain (physical well‐being domain).

Table 11 shows that the reported QoL of the children with DS on the psychological well‐being domain is “very good” with an average of (3.65 out of 5.00). This average falls in the fourth category of fifth scale categories in the scale of weighted mean for QoL table (from 3.4 to 4.2).

**TABLE 11:** Range of reported QoL in the domain of psychological well‐being.

Question	Count	%	Mean	Std. deviation	Rank	Range
Has your child felt that life was enjoyable?						
Not at all	5	4.5	3.4911	1.10686	7	Very good
Yes, but a little	18	16.1				
Average yes	27	24.1				
Well yes and acceptable	41	36.6				
Very yes	21	18.8				
Has your child been in a good mood?						
Not at all	3	2.7	3.5536	0.85781	4	Very good
Yes, rarely	7	6.3				
Yes, sometimes	38	33.9				
Often yes	53	47.3				
Yes, always	11	9.8				
Has your child had fun?						
Not at all	5	4.5	3.5357	1.01279	6	Very good
Yes, rarely	9	8.0				
Yes, sometimes	38	33.9				
Often yes	41	36.6				
Yes, always	19	17.0				
Has your child felt sad?						
Yes, always	2	1.8	3.5446	0.89933	5	Very good
Often yes	7	6.3				
Yes, sometimes	49	43.8				
Yes, rarely	36	32.1				
Not at all	18	16.1				
Has your child felt so bad that he/she did not want to do anything?						
Yes, always	1	0.9	3.6696	0.92404	2	Very good
Often yes	5	4.5				
Yes, sometimes	51	45.5				
Yes, rarely	28	25.0				
Not at all	27	24.1				
Has your child felt lonely?						
Yes, always	4	3.6	4.1071	1.09345	1	Very good
Often yes	4	3.6				
Yes, sometimes	25	22.3				
Yes, rarely	22	19.6				
Not at all	57	50.9				
Has your child been happy with the way he/she is?						
Not at all	7	6.3	3.6607	1.11140	3	Very good
Yes, rarely	8	7.1				
Yes, sometimes	28	25.0				
Often yes	42	37.5				
Yes, always	27	24.1				
Psychological well‐being			3.6518	0.73791	Very good	

The responses also indicated that the sixth question, “Has your child felt lonely?”, scored the highest in terms of QoL with an average of (4.1 out of 5). On the other hand, the first question, “Has your child felt that life was enjoyable?”, scored the least in terms of QoL with an average of (3.49 out of 5).

##### Third domain: Autonomy and parent relation

Table 12 shows that the reported QoL of the children with DS on the autonomy and parent relation domain is “very good” with an average of (3.72 out of 5.00). This average falls in the fourth category of fifth scale categories in the scale of weighted mean for QoL table (from 3.4 to 4.2).

**TABLE 12:** Range of reported QoL in the domain of autonomy and parent relation.

Question	Count	%	Mean	Std. deviation	Rank	Range
Has your child had enough time for him/herself?						
Not at all	2	1.8	3.7411	0.96558	3	Very good
Yes, rarely	9	8.0				
Yes, sometimes	31	27.7				
Often yes	44	39.3				
Yes, always	26	23.2				
Has your child been able to do the things that he/she wants to do in his/her free time?						
Not at all	0	0.0	3.6250	0.88149	5	Very good
Yes, rarely	10	8.9				
Yes, sometimes	42	37.5				
Often yes	40	35.7				
Yes, always	20	17.9				
Has your child felt that his/her parent(s) had enough time for him/her?						
Not at all	1	0.9	3.7857	0.94372	2	Very good
Yes, rarely	7	6.3				
Yes, sometimes	37	33.0				
Often yes	37	33.0				
Yes, always	30	26.8				
Has your child felt that his/her parent(s) treated him/her fairly?						
Not at all	0	0.0	4.1607	0.84420	1	Very good
Yes, rarely	2	1.8				
Yes, sometimes	26	23.2				
Often yes	36	32.1				
Yes, always	48	42.9				
Has your child been able to talk to his/her parent(s) when he/she wanted to?						
Not at all	5	4.5	3.7232	1.10045	4	Very good
Yes, rarely	7	6.3				
Yes, sometimes	36	32.1				
Often yes	30	26.8				
Yes, always	34	30.4				
Has your child had enough money to do the same things as his/her friends?						
Not at all	9	8.0	3.4911	1.25199	7	Very good
Yes, rarely	15	13.4				
Yes, sometimes	31	27.7				
Often yes	26	23.2				
Yes, always	31	27.7				
Has your child felt that he/she had enough money for his/her expenses?						
Not at all	10	8.9	3.5268	1.28020	6	Very good
Yes, rarely	14	12.5				
Yes, sometimes	28	25.0				
Often yes	27	24.1				
Yes, always	33	29.5				
Autonomy and parent relation domain			3.7219	0.63739	Very good	

The responses also indicated that the fourth question, “Has your child felt that his/her parent(s) treated him/her fairly?”, scored the highest in terms of QoL with an average of (4.16 out of 5). On the other hand, the sixth question, “Has your child had enough money to do the same things as his/her friends?”, scored the least in terms of QoL with an average of (3.49 out of 5).

##### Fourth domain: Social well‐being and peers

Table 13 shows that the reported QoL of the children with DS on the social well‐being and peers domain is “good” with an average of (2.79 out of 5.00). This average falls in the third category of fifth scale categories in the scale of weighted mean for QoL table (from 2.6 to 3.4).

**TABLE 13:** Range of reported QoL in the domain of social well‐being and peers.

Question	Count	%	Mean	Std. deviation	Rank	Range
Has your child spent time with his/her friends?						
Not at all	22	19.6	2.7143	1.13446	3	Good
Yes, rarely	21	18.8				
Yes, sometimes	41	36.6				
Often yes	23	20.5				
Yes, always	5	4.5				
Has your child had fun with his/her friends?						
Not at all	17	15.2	3.0000	1.22290	1	Good
Yes, rarely	20	17.9				
Yes, sometimes	33	29.5				
Often yes	30	26.8				
Yes, always	12	10.7				
Have your child and his/her friends helped each other?						
Not at all	18	16.1	2.9107	1.23430	2	Good
Yes, rarely	24	21.4				
Yes, sometimes	32	28.6				
Often yes	26	23.2				
Yes, always	12	10.7				
Has your child been able to rely on his/her friends?						
Not at all	27	24.1	2.5357	1.18496	4	Fair
Yes, rarely	28	25.0				
Yes, sometimes	34	30.4				
Often yes	16	14.3				
Yes, always	7	6.3				
Social well‐being and peers domain			2.7902	1.04329	Good	

The responses also indicated that the second question, “Has your child had fun with his/her friends?”, scored the highest in terms of QoL with an average of (3.00 out of 5). On the other hand, the fourth question, “Has your child been able to rely on his/her friends?”, scored the least in terms of QoL with an average of (2.53 out of 5).

##### Fifth domain: School and learning

Table 14 shows that the reported QoL of the children with DS on the school and learning domain is “very good” with an average of (4.17 out of 5.00). This average falls in the fourth category of fifth scale categories in the scale of weighted mean for QoL table (from 3.4 to 4.2).

**TABLE 14:** Range of reported QoL in the domain of school and learning.

Question	Count	%	Mean	Std. deviation	Rank	Range
Has your child been happy at school?						
Not at all	3	3.0	4.2525	0.99328	2	Excellent
Yes, rarely	4	4.0				
Yes, sometimes	9	9.1				
Often yes	32	32.3				
Yes, always	51	51.5				
Has your child got on well at school?						
Not at all	4	4.0	4.1515	0.90761	3	Very good
Yes, rarely	1	1.0				
Yes, sometimes	7	7.1				
Often yes	51	51.5				
Yes, always	36	36.4				
Has your child been able to pay attention?						
Not at all	0	0.0	3.6566	0.84710	4	Very good
Yes, rarely	5	5.1				
Yes, sometimes	43	43.4				
Often yes	32	32.3				
Yes, always	19	19.2				
Has your child got along well with his/her teachers?						
Not at all	0	0.0	4.6465	0.65952	1	Excellent
Yes, rarely	2	2.0				
Yes, sometimes	4	4.0				
Often yes	21	21.2				
Yes, always	72	72.7				
School and learning			4.1768	0.65001	Very good	

The responses also indicated that the fourth question, “Has your child got along well with his/her teachers?”, scored the highest in terms of QoL with an average of (4.64 out of 5). On the other hand, the third question, “Has your child been able to pay attention?”, scored the least in terms of QoL with an average of (3.65 out of 5).

The table shows the mean and standard deviation for five different domains of reported QoL for children with DS. The domains include physical well‐being, psychological well‐being, autonomy and parental relation, social well‐being and peers, and school and learning environment (Table 15).

**TABLE 15:** Ranges of children's quality of life on the five domains (physical well‐being, psychological well‐being, autonomy and parent relation, social well‐being and peers, and school and learning environment) (as summary).

Domain	Mean	Std. deviation	Reported QoL of children with DS
Physical well‐being	3.35	0.78	Good
Psychological well‐being	3.65	0.73	Very good
Autonomy and parental relation	3.72	0.63	Very good
Social well‐being and peers	2.79	1.04	Good
School and learning environment	4.17	0.65	Very good

The results indicate that the highest mean score for QoL was reported in the domain of school and learning environment (4.17), which was rated as “very good.” The second highest mean score was reported in the domain of autonomy and parental relation (3.72), which was also rated as “very good.” The psychological well‐being of the children was also rated as “very good” with a mean score of (3.65).

On the other hand, the social well‐being and peers domain had the lowest mean score of (2.79), which was still rated as “good.” It is worth noting that this domain also had the highest standard deviation of (1.04), indicating greater variability in the responses for this domain.

The table provides the results of a study that investigated the relationship between different variables (parental age, parental education level, nationality, Financial Status, and Child's Gender) and various domains of well‐being in children. The domains of well‐being include physical well‐being, psychological well‐being, autonomy and parent relation, social well‐being and peers, and school and learning environment. The study used Kruskal–Wallis *H* test to determine if there are any significant differences in mean ranks across the categories of each variable. For each domain, the table shows the number of participants (*N*), the mean rank of each group, the percentage of each group, the Kruskal–Wallis *H* statistic, and the significance level (Sig). The findings suggest that parental age has a significant effect on children's psychological well‐being, with older parental age associated with higher mean ranks. However, parental age did not have a significant effect on the other domains of well‐being. Parental education level did not have a significant effect on any of the domains of well‐being, although there were some trends suggesting that higher education levels were associated with higher mean ranks in some domains.

The results showed that there were no significant differences between Saudi and non‐Saudi children in any of the domains. However, significant differences were observed in the physical well‐being, psychological well‐being, autonomy and parent relation, social well‐being and peers, and school and learning environment domains based on financial status. Specifically, children with a financial status of SAR 3000 or less had significantly lower mean ranks in all domains compared to children with higher financial statuses.

In terms of gender, there were no significant differences in any of the domains between male and female children in the physical well‐being, psychological well‐being, autonomy and parent relation, and social well‐being and peers domains. However, in the school and learning environment domain, female children had significantly higher mean ranks compared with male children.

Overall, the study highlights the significant impact of financial status on child development, particularly in the domains of physical well‐being, psychological well‐being, autonomy and parent relation, social well‐being and peers, and school and learning environment. Gender also appears to play a role in child development, specifically in the school and learning environment domain.

## 4 DISCUSSION

DS is a common chromosomal disorder that is associated with a variety of attributes and challenges. These complications call for early interventions that are tailored to each individual's needs. Caring for a child with DS can pose various challenges because children with this disorder need special handling physically, mentally, emotionally, and financially, requiring guided knowledge and support to facilitate the parental approach to meet the variety of needs arising in the child's life. These different needs require skilled consideration from professionals in health care, education, and the community. Differences in demographic variables affect the QoL of a child with DS.

This study emerged after the researcher observed variations in the QoL of children with DS, in relation to demographic data of parents and their children who visited the Genetics Clinic, which the researcher also attended. We focused on detecting statistical significance of the demographic data on the reported QoL of DS children. As such, this study of demographic variables will introduce an understanding of the existing situation for children with DS and informatively guiding future interventions to effectively serve children and families, reducing the gap between the needed and available services.

Parents' reports of their children's QoL ranged from “good” to “very good” in all five domains. The physical well‐being domain was positive significantly influenced by family income level. The psychological well‐being domain was positive significantly influenced by family income and parental age. The domain of autonomy and parent relations was not influenced by any variable other than family income. The highest QoL was observed in the group of parents older than 40 years of age, and the lowest QoL was reported by the group under 30 years of age.

### 4.1 The children's characteristics and their parents' preliminary demographic data

Participants' demographic data reveals that more mothers (76.8%) than fathers (23.2%) participated in the study. This finding is similar to that of Duggan et al. (2015), who found that mothers are more likely than fathers to engage with social media outlets to receive and provide parenting‐related information and support. This conclusion could also explain the lack of participation among individuals who were approached via certain non‐governmental organizations that target children with DS in the more conservative cities such as Jazan, Albaha, Najran, Tabuk, and Al‐Jawf. Such a trend is especially understandable given the Saudi culture's unease with communication between genders and the tendency for mothers to be more involved than fathers in childcare (Al‐Saggaf, 2016). The gender of the researcher (the researcher's gender is male) may have also contributed to this finding, as he was informed that certain social media groups were for women only, and he could not be added to those groups because of his gender. The areas of Jazan, Albaha, Najran, Tabuk, and Al Jawf are conservative compared to the more metropolitan communities of major cities (e.g., Eastern Province, Riyadh, Jeddah, and Mecca).

The study investigated the age of the mothers of our participants. Despite a known higher incidence of Ds in pregnant mothers >40, more infants are typically conceived at younger ages so most mothers with Ds are also younger. This was seen in in our study as well where 94 out of 112 participants with children with Ds were <40. This pattern is consistent with the National DS Society's postulation from 2020, which indicates that a higher birth rate among younger women results in approximately 80% of DS children being born to mothers under the age of 35 years. (NDSS, 2020).

### 4.2 Range of children's QoL

Parental reports of the QoL of their children with DS ranged from “good” to “very good” in all five domains. These findings are somewhat similar to the findings of the Australian study on children with DS using the same tool. In that study, the children scored within normal ranges in all domains, excepts in physical and social well‐being (Rofail et al., 2017).

### 4.3 Influence of children with DS and their parents' demographic variables on each of the five QoL these children's domains

#### 4.3.1 Physical well‐being

The physical well‐being domain was significantly influenced by family income level. The lack of influence of other demographic variables on physical well‐being was surprising, as other studies demonstrated that parental education influences the children's health (Diaz, 2020). This study shows how the highest QoL in the physical well‐being domain was observed by a participant with less than primary school education, while the second highest was observed by individuals with only a primary school education.

#### 4.3.2 Psychological well‐being

This domain was significantly influenced by family income and parental age. The family income level significantly influenced the psychological well‐being of the children, agreeing with the China study that concluded that family wealth has a favorable impact on children's emotional well‐being outcomes such as depression, hopelessness, helplessness, and meaninglessness. Second, the data reveals that family wealth is highly connected with parents' emotional well‐being, which affects children's well‐being (Qi & Wu, 2020).

The majority (60.7%) of participating parents were older than 40, and the highest QoL was present in this age group. Meanwhile, the lowest QoL was reported by the group under 30 years of age. This contradicts previous research that found no psychosocial advantage or disadvantage in relation to maternal age group and no impact of maternal age on emotional or behavioral well‐being in early and middle childhood (Boivin et al., 2009). The connection between higher QoL and advanced parental age could be attributed to the accumulation of life experience with age, as this usually enhances a person's ability to cope with life stressors, especially social stigma, specifically for parents of children with special needs (Tilahun et al., 2016).

#### 4.3.3 Autonomy and parent relations

This domain was not influenced by any variable other than family income. It has a positive correlation with high income, which may be attributable to insufficient community awareness, child and family factors, and autonomy for the DS child (Gilmore et al., 2016). When applied to the SA context, family dynamics generally do not support children's autonomy. According to the World Health Organization (2010), it is crucial to empower children and young people with intellectual disabilities who contribute to decision‐making in their lives and autonomy (“Better health, better lives”) (World Health Organization, 2010).

#### 4.3.4 Social well‐being and peers

This domain was significantly influenced by parental education. Higher education was linked to better social QoL. This could be attributed to parents with a high level of education being aware of the importance of friends and social sharing in increasing their children's cognitive abilities.

This is in accordance with that of Assari and Caldwell (2019) who identified a positive correlation between parental education and the children's QoL, in which a higher parental education corresponded to a higher degree of social QoL (Currie, 2020). In addition, Erola et al. (2016) argued that parental education is critical in predicting the educational and social lives of their children (Erola et al., 2016). In contrast, Jung and Lee (2017) added regarding the child's functioning, which reflects on participation to factors influencing the child with DS QoL. It was noted that this domain was the only one not influenced by family income level (Jung & Lee, 2017).

#### 4.3.5 The school and learning environment domain

It is significantly influenced by the child's gender. Results of this study suggest that females tend to report a better school environment than males, in agreement with a Turkish study that recorded higher job satisfaction among female teachers at the early childhood level. Job satisfaction of female teachers may be related to more enjoyment of the school environment with female children than their male counterparts (Şahin & Sak, 2016). Another study stated that female teachers formed close relationships with female students, but female teachers were less accepted by male students. However, male and female teachers had more difficulties in relationships with boys than with girls. (Spilt et al., 2012). This variation in school experience, based on the child's gender, might also be present in SA schools, implying a better school environment for female than male students. Furthermore, it is worth noting that in SA, schools are typically segregated by gender. Boys and girls attend separate educational institutions, except during the initial three stages of primary education.

The school and learning environment registered the highest QoL in the income group earning SAR 3000 or less, which is roughly equivalent to 800 USD. This warrants investigation and may be explained by how families with lower income experience greater appreciation for free services (Zun et al., 2018). Although there are few DS government schools and they are located only in the main cities, people with limited income are usually dependent on these schools, which may be more efficient than private schools used by high‐income families. This theory is one possible explanation, and we agree with this, as we observed in our DS clinic that most of our patients studying in government schools were more active and cognitive than children in private schools. That may be attributed to the great interest in this category from the Saudi education ministry, government agencies such as the health ministry and community affairs ministry, and the financial and logistical support from the community, which are directed directly to developing and creating a comfortable and healthy environment in their government schools. However, many private schools in major cities compete with government schools in terms of quality and the active and cognitive abilities of their students, such as the “Help Centre” in Jeddah. As observed in our clinic, the cognitive.

Abilities of students at the “Help Center” in Jeddah are exceptional. This has also been indicated in some studies that suggest the quality of private education surpasses that of public education (Chordia et al., 2020; Das, 2017; Kingdon, 2020).

### 4.4 Highlights

All QoL of children with DS was influenced by family income level except social well‐being and peer domain. Physical and psychological well‐being were positively correlated with income, while autonomy and parent relation were negatively correlated. These findings indicate that a higher income can be linked to a higher QoL (Table 16). This agrees with a previous study, suggesting a significant relationship between family income and acceptance of intervention, influencing the families' understandings of the strengths, abilities, and special needs of children with DS (Mohammed Nawi et al., 2013).

**TABLE 16:** Influence of each demographic variables (parental age, parental education level, nationality, financial status, and child gender) on the five domains of quality of life (physical well‐being, psychological well‐being, autonomy and parent relation, social well‐being and peers, and school and learning environment).

Variable	Domain	Category	*N*	Mean rank	%	Kruskal–Wallis H	*p*‐Value	Sig/domain
Parental Age	Physical well‐being	Less than 30 years	21	50.5	30.30	0.89	0.64	N.Sig.
		From 30 to 40 years	23	58.13	34.90			
		More than 40 years	68	57.8	34.70			
	Psychological well‐being	Less than 30 years	21	39.98	25.60	8.21	0.01	Sig.
		From 30 to 40 years	23	53.2	34.10			
		More than 40 years	68	62.72	40.20			
	Autonomy and parent relation	Less than 30 years	21	50.38	30.00	1.15	0.56	N.Sig.
		From 30 to 40 years	23	60.74	36.10			
		More than 40 years	68	56.96	33.90			
	Social well‐being and peers	Less than 30 years	21	65.5	37.50	2.01	0.36	N.Sig.
		From 30 to 40 years	23	55.11	31.50			
		More than 40 years	68	54.19	31.00			
	School and learning environment	Less than 30 years	20	48.78	33.10	0.31	0.85	N.Sig.
		From 30 to 40 years	19	47.39	32.20			
		More than 40 years	60	51.23	34.80			
Parental Education Level	Physical well‐being	Less than primary	1	110.5	28.30	4.70	0.45	N.Sig.
		Primary	1	68.5	17.50			
		Prep.	9	43.5	11.10			
		Secondary	25	54.24	13.90			
		Bachelor	67	58.23	14.90			
		Postgraduate	9	55.56	14.20			
	Psychological well‐being	Less than primary	1	105.5	33.10	9.18	0.10	N.Sig.
		Primary	1	6	1.90			
		Prep.	9	39.28	12.30			
		Secondary	25	51.2	16.10			
		Bachelor	67	60.93	19.10			
		Postgraduate	9	55.67	17.50			

	Autonomy and parent relation	Less than primary	1	90.5	22.60	6.10	0.29	N.Sig.
		Primary	1	64	16.00			
		Prep.	9	61.78	15.40			
		Secondary	25	63.2	15.80			
		Bachelor	67	50.84	12.70			
		Postgraduate	9	70.11	17.50			
	Social well‐being and peers	Less than primary	1	99	27.00	12.44	0.02	Sig.
		Primary	1	47	12.80			
		Prep.	9	34.72	9.50			
		Secondary	25	48	13.10			
		Bachelor	67	59.07	16.10			
		Postgraduate	9	79.11	21.60			
	School and learning environment	Less than primary	1	91.5	27.20	4.61	0.46	N.Sig.
		Primary	1	31.5	9.40			
		Prep.	9	60.17	17.90			
		Secondary	24	48.5	14.40			
		Bachelor	56	47.66	14.20			
Postgraduate		8	56.56	16.80				
Nationality	Physical well‐being	Saudi	97	58	55.30	582	0.21	N.Sig.
		Non‐Saudi	15	46.8	44.70			
	Psychological well‐being	Saudi	97	57.34	52.90	646.5	0.48	N.Sig.
		Non‐Saudi	15	51.1	47.10			
	Autonomy and parent relation	Saudi	97	57.63	53.90	618	0.34	N.Sig.
		Non‐Saudi	15	49.2	46.10			
	Social well‐being and peers	Saudi	97	58.06	55.60	576.5	0.19	N.Sig.
		Non‐Saudi	15	46.43	44.40			
	School and learning environment	Saudi	85	49.68	48.90	567.5	0.77	N.Sig.
		Non‐Saudi	14	51.96	51.10			
Financial Status (Saudi Arabian Riyal)	Physical well‐being	SAR 3000 or less	8	32.25	16.00	9.95	0.01	Sig.
		SAR 3001–6000	25	57.68	28.50			
		SAR 6001–10,000	28	47.5	23.50			
		More than SAR 10,000	51	64.67	32.00			
	Psychological well‐being	SAR 3000 or less	8	15.56	8.30	19.68	<0.01	Sig.
		SAR 3001–6000	25	46.5	24.70			
		SAR 6001–10,000	28	60.3	32.10			
		More than SAR 10,000	51	65.74	34.90			
	Autonomy and parent relation	SAR 3000 or less	8	35.5	17.20	8.18	0.04	Sig.
		SAR 3001–6000	25	46.14	22.40			
		SAR 6001–10,000	28	63.05	30.60			
		More than SAR 10,000	51	61.27	29.70			
	Social well‐being and peers	SAR 3000 or less	8	29.75	14.50	6.10	0.10	N.Sig.
		SAR 3001–6000	25	56.42	27.50			
		SAR 6001–10,000	28	60.5	29.50			
		More than SAR 10,000	51	58.54	28.50			
	School and learning environment	SAR 3000 or less	8	81.5	36.70	13.28	0.00	Sig.
		SAR 3001–6000	24	39.75	17.90			
		SAR 6001–10,000	24	52.21	23.50			
		More than SAR 10,000	43	48.63	21.90			
Child's Gender	Physical well‐being	Female	53	53.38	47.40	1398	0.33	N.Sig.
		Male	59	59.31	52.60			
	Psychological well‐being	Female	53	55.55	49.20	1513	0.76	N.Sig.
		Male	59	57.36	50.80			
	Autonomy and parent relation	Female	53	50.86	45.20	1264.5	0.08	N.Sig.
		Male	59	61.57	54.80			
	Social well‐being and peers	Female	53	52.91	47.00	1373	0.26	N.Sig.
		Male	59	59.73	53.00			
	School and learning environment	Female	47	57.39	57.00	874.5	0.01	Sig.
		Male	52	43.32	43.00			

*Note*: Sig *α* = 0.05 sig. = significant N.Sig. = nonsignificant.

Demographic races and nationality did not influence all QoL domains, might be attributed to equality in government and private services for Saudis and non‐Saudi people.

### 4.5 Limitations

The study has several limitations that should be considered. Firstly, parental reports of their children's QoL can vary and may not fully capture the child's experience. Secondly, using a questionnaire for data collection introduces the possibility of self‐reporting bias, where participants may provide answers that reflect their beliefs or preferences rather than their actual experiences. Thirdly, the researcher's gender may have limited the participation of mothers in the study in the more conservative cities such as Jazan, Albaha, Najran, Tabuk, and Al‐Jawf, potentially limiting the study's sample and generalizability to all citizens in SA. Fifthly, the authors did not consider an aspect as important as the degree of intellectual disability in DS. Additionally, the study may have included mostly caring parents who are more involved in their child's life than those who are less concerned. Furthermore, demographic data related to a child's education (whether governmental or private) were not collected, despite its potential to affect the child's QoL. Finally, since the results were not corrected for multiple comparisons, the findings should be interpreted as exploratory and hypothesis‐generating rather than definitive or conclusive.

## 5 CONCLUSION

This study explores the relationship between demographic variables of children with DS and their parents and the QoL of these children in SA. The discussion provides a detailed analysis of the study's findings and their implications. The results show that family income level had a significant positive influence on the physical well‐being domain, while both parental age and family income level had a significant positive influence on the psychological well‐being domain. However, the autonomy and parent relations domain were not significantly influenced by any variable other than family income. The study also reveals a higher participation rate of mothers than fathers The QoL of children with DS was found to range from “good” to “very good” in all five domains. The study's findings have significant implications for early interventions tailored to each individual's needs, the guidance and support required by parents, and the role of professionals in healthcare, education, and the community. These findings emphasize the significance of considering gender‐related factors when conducting research in specific geographic areas, highlighting the necessity for more inclusive research practices. Furthermore, the study underscores the relevance of these variables for genetic counselors involved in managing children with DS.

## AUTHOR CONTRIBUTIONS

**Nuha Alrayes, Omar Y. Alghubayshi, and Jumana Y. Al‐Amaa:** Conceptualization. **Omar Y. Alghubayshi and Nuha Alrayes:** Data collection. **Nuha Alrayes, Omar Y. Alghubayshi, Ashwaq Hassan Alsabban, and Noha M. Issa:** Data analysis. **Nuha Alrayes:** Funding acquisition. **Noha M. Issa, Omar Y. Alghubayshi, Jumana Y. Al‐Amaa, Reem Abdullah Alyoubi, and Yaser M. Alkhiary:** Methodology. **Nuha Alrayes and Yaser M. Alkhiary:** Project administration and supervision. **Nuha Alrayes, Dalal Sameer Al Shaer, Reem Abdullah Alyoubi, Khalidah K. Nasser, and Noha M. Issa:** Resources. **Nuha Alrayes, Omar Y. Alghubayshi, Noha M. Issa, Jumana Y. Al‐Amaa, Ashwaq Hassan Alsabban, Dalal Sameer Al Shaer, Reem Abdullah Alyoubi, Khalidah K. Nasser, and Yaser M. Alkhiary:** Writing original draft and review.

## FUNDING INFORMATION

Ministry of Education and King Abdulaziz University, Jeddah, Saudi Arabia, Grant no. (IFPRC‐050‐142‐2020).

## CONFLICT OF INTEREST STATEMENT

The authors declare no conflict of interest for this article.

## Supporting information


Appendix S1.

## Data Availability

All datasets analyzed for this study are included in the article.
