# Supplementary material for: Quality of life in children with Down syndrome and its association with parent and child demographic characteristics: Parent‐reported measures
Source: Mol Genet Genomic Med. 2023 Dec 13;12(1):e2337. doi: 10.1002/mgg3.2337 (PMC10767681; doi:10.1002/mgg3.2337)
Supplement: Supplementary file 1 — Appendix S1. [file MGG3-12-e2337-s001.docx]

**Appendix 1 (Arabic version of the survey)**

**أولًا: الخصائص الشخصية للمبحوثين:**

1. صلة القرابة للطفل:

- الأب ( )
- الأم ( )

أخرى تذكر................. .

1. العمر: .......................
2. الجنسية:

- سعودي
- غير سعودي

1. منطقة السكن

- محافظة جدة
- منطقة مكة المكرمة خارج محافظة جدة
- منطقة المدينة المنورة
- منقطة القصيم
- المنطقة الشرقية
- منطقة عسير
- منطقة تبوك
- منطقة حائل
- منطقة الحدود الشمالية
- منطقة جازان
- منطقة نجران
- منطقة الباحة
- منطقة الجوف
- منطقة الرياض
- اسم المدينة ( لمن يسكن خارج محافظة جدة ) ....................

1. مستوى التعليم:

- أقل من ابتدائي
- ابتدائي
- متوسط
- ثانوي
- جامعي
- دراسات عليا

1. مستوى دخل الأسرة

- 3000 ريال وأقل
- من 3001 الى 6000 ريال
- من 6001 إلى 10000 ريال
- أعلى من 10001 ريال

**ثانيًا: الخصائص الشخصية لأطفال داون**

1. عدد أطفال داون داخل الأسرة:

- طفل واحد
- أخرى

1. جنس الطفل:

- ذكر
- انثى

1. عمر الطفل: ................. .
2. عمر الأم عند إنجاب الطفل:

- 30 سنه وأقل
- 31 سنة – 35 سنة
- ٣٦ – الى ٤٠ سنة
- 41 سنه وأكثر

**Kidscreen 27- for parents (Arabic version)**

**تقييم محور الأنشطة البدنية والصحية للطفل : ( فكر في اخر أسبوع )**

**الأنشطة البدنية والصحة**

1. بشكل عام، كيف يقيم ابنك صحته؟

| ممتازة | جيدة جدا | جيدة | مقبولة | ضعيفة |
| --- | --- | --- | --- | --- |
|  |  |  |  |  |

**فكر في الأسبوع الماضي...**

1. هل شعر طفلك بأنه لائق وبخير؟

| لا على الإطلاق | نعم - قليلاً | نعم – متوسط | نعم - جداً | نعم – للفاية |
| --- | --- | --- | --- | --- |
|  |  |  |  |  |

1. هل كان طفلك نشيطاً بدنياً؟ (مثال الجري، التسلق، ركوب الدراجات)

| لا على الإطلاق | نعم - قليلاً | نعم – متوسط | نعم - جداً | نعم – للفاية |
| --- | --- | --- | --- | --- |
|  |  |  |  |  |

1. هل كان طفلك قادراً على الجري بشكل جيد

| لا على الإطلاق | نعم - قليلاً | نعم – متوسط | نعم - جداً | نعم - للفاية |
| --- | --- | --- | --- | --- |
|  |  |  |  |  |

**فكر في الأسبوع الماضي...**

1. هل شعر طفلك بأنه مليء بالطاقة؟

| لا على الاطلاق | نعم نادراً | نعم أحياناً | نعم غالباً | نعم دائماً |
| --- | --- | --- | --- | --- |
|  |  |  |  |  |

**الحالة النفسية والمزاج العام والمشاعر**

**فكر في الأسبوع الماضي...**

1. هل شعر طفلك بأن حياته ممتعة؟

| لا على الإطلاق | نعم ولكن قليلاً | نعم بشكل متوسط | نعم بشكل جيد ومقبول | نعم للفاية |
| --- | --- | --- | --- | --- |
|  |  |  |  |  |

**فكر في الأسبوع الماضي...**

1. هل كان طفلك في مزاج جيد؟

| لا مطلقاً | نعم نادراً | نعم أحياناً | نعم غالباً | نعم دائماً |
| --- | --- | --- | --- | --- |
|  |  |  |  |  |
|  |  |  |  |  |

1. هل حصل طفلك على المتعة؟

| لا مطلقاً | نعم نادراً | نعم أحياناً | نعم غالباً | نعم دائماً |
| --- | --- | --- | --- | --- |
|  |  |  |  |  |

**فكر في الأسبوع الماضي...**

1. هل شعر طفلك بالحزن؟

| لا مطلقاً | نعم نادراً | نعم أحياناً | نعم غالباً | نعم دائماً |
| --- | --- | --- | --- | --- |
|  |  |  |  |  |

1. هل شعر طفلك بالاستياء لدرجة انه لم يكن يريد فعل أي شيء؟

| لا مطلقاً | نعم نادراً | نعم أحياناً | نعم غالباً | نعم دائماً |
| --- | --- | --- | --- | --- |
|  |  |  |  |  |

1. هل شعر طفلك بالوحدة؟

| لا مطلقاً | نعم نادراً | نعم أحياناً | نعم غالباً | نعم دائماً |
| --- | --- | --- | --- | --- |
|  |  |  |  |  |

1. هل كان طفلك يشعر بالسعادة بالطريقة التي كان يبدو عليها؟

| لا مطلقاً | نعم نادراً | نعم أحياناً | نعم غالباً | نعم دائماً |
| --- | --- | --- | --- | --- |
|  |  |  |  |  |

**العائلة ووقت الفراغ**

**فكر في الأسبوع الماضي...**

1. هل كان لدى طفلك الوقت الكافي لنفسه؟

| لا على الاطلاق | نعم نادراً | نعم أحياناً | نعم غالباً | نعم دائماً |
| --- | --- | --- | --- | --- |
|  |  |  |  |  |

1. هل كان طفلك قادراً على فعل الأشياء التي يود القيام بها في وقت فراغه؟

| لا على الاطلاق | نعم نادراً | نعم أحياناً | نعم غالباً | نعم دائماً |
| --- | --- | --- | --- | --- |
|  |  |  |  |  |

1. هل شعر طفلك أن لدى والديه وقتاً كافٍ له؟

| لا على الاطلاق | نعم نادراً | نعم أحياناً | نعم غالباً | نعم دائماً |
| --- | --- | --- | --- | --- |
|  |  |  |  |  |

1. هل يشعر طفلك أن والديه يعاملانه بإنصاف؟

| لا على الاطلاق | نعم نادراً | نعم أحياناً | نعم غالباً | نعم دائماً |
| --- | --- | --- | --- | --- |
|  |  |  |  |  |

1. هل كان طفلك قادراً على التكلم مع والديه عندما يريد ذلك؟

| لا على الاطلاق | نعم نادراً | نعم أحياناً | نعم غالباً | نعم دائماً |
| --- | --- | --- | --- | --- |
|  |  |  |  |  |

1. هل كان لدى طفلك المال الكافي لفعل نفس الأشياء التي يفعلها أصدقاؤه؟

| لا على الاطلاق | نعم نادراً | نعم أحياناً | نعم غالباً | نعم دائماً |
| --- | --- | --- | --- | --- |
|  |  |  |  |  |

1. هل شعر طفلك ان لديه المال الكافي لمصاريفه؟

| لا على الاطلاق | نعم نادراً | نعم أحياناً | نعم غالباً | نعم دائماً |
| --- | --- | --- | --- | --- |
|  |  |  |  |  |

**الاصدقاء** ) فكر في الأسبوع الماضي...(

1. هل قضى طفلك وقتاً مع الاصدقاء؟

| لا على الاطلاق | نعم نادراً | نعم أحياناً | نعم غالباً | نعم دائماً |
| --- | --- | --- | --- | --- |
|  |  |  |  |  |

1. هل حصل طفلك على المتعة مع اصدقائه؟

| لا على الاطلاق | نعم نادراً | نعم أحياناً | نعم غالباً | نعم دائماً |
| --- | --- | --- | --- | --- |
|  |  |  |  |  |

1. هل ساعد طفلك وأصدقاؤه بعضهم البعض؟

| لا على الاطلاق | نعم نادراً | نعم أحياناً | نعم غالباً | نعم دائماً |
| --- | --- | --- | --- | --- |
|  |  |  |  |  |

1. هل كان طفلك قادراُ على الاعتماد على اصدقائه؟

| لا على الاطلاق | نعم نادراً | نعم أحياناً | نعم غالباً | نعم دائماً |
| --- | --- | --- | --- | --- |
|  |  |  |  |  |

**المدرسة والتعليم**

**فكر في الأسبوع الماضي...( لا تقم بتعبئتها - عند اختيارك ابني غير منتسب لأي جهة تعليمية )**

1. هل كان طفلك سعيداً في المدرسة؟

| لا على الإطلاق | نعم – نادرا | نعم أحيانا | نعم غالبا | نعم دائما |
| --- | --- | --- | --- | --- |
|  |  |  |  |  |

1. هل كان طفلك على ما يرام في المدرسة؟

| لا على الإطلاق | نعم – نادرا | نعم أحيانا | نعم غالبا | نعم دائما |
| --- | --- | --- | --- | --- |
|  |  |  |  |  |

**فكر في الأسبوع الماضي...**

1. هل كان طفلك قادراً على الانتباه؟

| مطلقاً | نعم – نادرا | نعم أحيانا | نعم غالبا | نعم دائما |
| --- | --- | --- | --- | --- |
|  |  |  |  |  |

1. هل كان طفلك على علاقات جيدة مع المعلمين؟

| مطلقاً | نعم – نادرا | نعم أحيانا | نعم غالبا | نعم دائما |
| --- | --- | --- | --- | --- |
|  |  |  |  |  |

**Appendix 2**

**Firstly: Personal Characteristics of Participants:**

1- Relationship to the Child:

- Father ( )
- Mother ( )
- Other: _________________

2- Age: _________________

3- Nationality:

- Saudi
- Non-Saudi

4- Residence:

- Jeddah Governorate
- Makkah Region outside of Jeddah Governorate
- Al-Madinah Al-Munawwarah Region
- Al-Qassim Region
- Eastern Province
- Aseer Region
- Tabuk Region
- Hail Region
- Northern Borders Region
- Jazan Region
- Najran Region
- Al-Baha Region
- Al-Jouf Region
- Riyadh Region
- City Name (for those living outside Jeddah Governorate): _________________

5- Education Level:

- Less than Primary School
- Primary School
- Intermediate
- Secondary
- University
- Postgraduate Studies

6- Family Income Level:

- 3000 Saudi Riyals or less
- From 3001 to 6000 Saudi Riyals
- From 6001 to 10000 Saudi Riyals
- Higher than 10001 Saudi Riyals

**Secondly: Personal Characteristics of Children with Down Syndrome**

1- Number of Children with Down Syndrome in the Family:

- One child
- Other: _________________

2- Gender of the Child:

- Male
- Female

3- Age of the Child: _________________

4- Age of the mother at the time of the child's birth:

- 30 years or less
- 31-35 years
- 36-40 years
- 41 years or more

**Kidscreen 27- for parents**

**1-Physical and Health well-being:**

1. Overall, how would you rate your child's health?

| Yes, extremely | very much | Yes, moderately | Yes, a little | Not at all |
| --- | --- | --- | --- | --- |
|  |  |  |  |  |

Think about last week...

2- Did your child feel fit and well?

| Yes, extremely | very much | Yes, moderately | Yes, a little | Not at all |
| --- | --- | --- | --- | --- |
|  |  |  |  |  |

3- Was your child physically active? (e.g. running, climbing, biking)

| Yes, extremely | very much | Yes, moderately | Yes, a little | Not at all |
| --- | --- | --- | --- | --- |
|  |  |  |  |  |

4- Was your child able to run well?

| Yes, extremely | very much | Yes, moderately | Yes, a little | Not at all |
| --- | --- | --- | --- | --- |
|  |  |  |  |  |

Think about last week...

5- Did your child feel energetic?

| Yes, extremely | very much | Yes, moderately | Yes, a little | Not at all |
| --- | --- | --- | --- | --- |
|  |  |  |  |  |

**2-Psychological well-being:**

Think about last week...

6- Did your child feel that their life was enjoyable?

| Yes, extremely | very much | Yes, moderately | Yes, a little | Not at all |
| --- | --- | --- | --- | --- |
|  |  |  |  |  |

Think about last week...

7- Was your child in a good mood?

| Yes, extremely | very much | Yes, moderately | Yes, a little | Not at all |
| --- | --- | --- | --- | --- |
|  |  |  |  |  |

8- Did your child have fun?

| Yes, extremely | very much | Yes, moderately | Yes, a little | Not at all |
| --- | --- | --- | --- | --- |
|  |  |  |  |  |

Think about last week...

9- Did your child feel sad?

| Yes, extremely | very much | Yes, moderately | Yes, a little | Not at all |
| --- | --- | --- | --- | --- |
|  |  |  |  |  |

10- Did your child feel so annoyed that they didn't want to do anything?

| Yes, extremely | very much | Yes, moderately | Yes, a little | Not at all |
| --- | --- | --- | --- | --- |
|  |  |  |  |  |

11- Did your child feel lonely?

| Yes, extremely | very much | Yes, moderately | Yes, a little | Not at all |
| --- | --- | --- | --- | --- |
|  |  |  |  |  |

12- Did your child feel happy in the way that they appeared to be?

| Yes, extremely | very much | Yes, moderately | Yes, a little | Not at all |
| --- | --- | --- | --- | --- |
|  |  |  |  |  |

3-**Autonomy and parent relation:**

Think about last week...

13- Did your child have enough time for themselves?

| Yes, extremely | very much | Yes, moderately | Yes, a little | Not at all |
| --- | --- | --- | --- | --- |
|  |  |  |  |  |

14- Was your child able to do the things they wanted to do in their free time?

| Yes, extremely | very much | Yes, moderately | Yes, a little | Not at all |
| --- | --- | --- | --- | --- |
|  |  |  |  |  |

15- Did your child feel that their parents had enough time for them?

| Yes, extremely | very much | Yes, moderately | Yes, a little | Not at all |
| --- | --- | --- | --- | --- |
|  |  |  |  |  |

16- Does your child feel that they are treated fairly by their parents?

| Yes, extremely | very much | Yes, moderately | Yes, a little | Not at all |
| --- | --- | --- | --- | --- |
|  |  |  |  |  |

17- Was your child able to talk to their parents when they wanted to?

| Yes, extremely | very much | Yes, moderately | Yes, a little | Not at all |
| --- | --- | --- | --- | --- |
|  |  |  |  |  |

18- Does your child have enough money to do the same things as their friends?

| Yes, extremely | very much | Yes, moderately | Yes, a little | Not at all |
| --- | --- | --- | --- | --- |
|  |  |  |  |  |

19- Does your child feel that they have enough money for their expenses?

| Yes, extremely | very much | Yes, moderately | Yes, a little | Not at all |
| --- | --- | --- | --- | --- |
|  |  |  |  |  |

**4- Social well- being and peers:**

Think about last week...

20- Did your child spend time with friends?

| Yes, extremely | very much | Yes, moderately | Yes, a little | Not at all |
| --- | --- | --- | --- | --- |
|  |  |  |  |  |

21- Did your child have fun with friends?

| Yes, extremely | very much | Yes, moderately | Yes, a little | Not at all |
| --- | --- | --- | --- | --- |
|  |  |  |  |  |

22- Did your child and their friends help each other?

| Yes, extremely | very much | Yes, moderately | Yes, a little | Not at all |
| --- | --- | --- | --- | --- |
|  |  |  |  |  |

23- Was your child able to rely on their friends?

| Yes, extremely | very much | Yes, moderately | Yes, a little | Not at all |
| --- | --- | --- | --- | --- |
|  |  |  |  |  |

**5- School and Learning environment:**

Think about last week...(Do not fill it out - if you have chosen my child is not enrolled in any educational institution)

24- Was your child happy at school?

| Yes, extremely | very much | Yes, moderately | Yes, a little | Not at all |
| --- | --- | --- | --- | --- |
|  |  |  |  |  |

25- Was your child doing well at school?

| Yes, extremely | very much | Yes, moderately | Yes, a little | Not at all |
| --- | --- | --- | --- | --- |
|  |  |  |  |  |

Think about last week...

26- Was your child able to pay attention?

| Yes, extremely | very much | Yes, moderately | Yes, a little | Not at all |
| --- | --- | --- | --- | --- |
|  |  |  |  |  |

27- Did your child have good relationships with their teachers?

| Yes, extremely | very much | Yes, moderately | Yes, a little | Not at all |
| --- | --- | --- | --- | --- |
|  |  |  |  |  |
